# Tuberculous Endophthalmitis With Phthisis Bulbi: A Diagnostic Delay Case Report

**DOI:** 10.1002/ccr3.72589

**Published:** 2026-04-29

**Authors:** Yanchen Chen, Shengnan Li, Xin Chen, Ningpu Liu, Dongjing Liu

**Affiliations:** ^1^ Department of Fundus Disease Sichuan Eye Hospital, AIER Eye Hospital Group Chengdu China

**Keywords:** diagnostic delay, endogenous endophthalmitis, miliary tuberculosis, *Mycobacterium tuberculosis*, ocular tuberculosis, phthisis bulbi

## Abstract

Tuberculous endophthalmitis should be prioritized in differential diagnosis for unexplained endophthalmitis in TB‐endemic regions; early systemic evaluation, timely pars plana vitrectomy, and adjunctive corticosteroids are crucial to prevent phthisis bulbi, though evidence for some interventions remains limited.

## 1 Introduction

Tuberculous endophthalmitis represents a rare yet devastating manifestation of ocular tuberculosis, frequently posing diagnostic challenges due to its nonspecific clinical features that overlap with other infectious or inflammatory conditions [1, 2]. Delayed diagnosis and suboptimal management commonly precipitate severe visual loss, occasionally necessitating enucleation, and consistently portend a grave prognosis [3, 4]. This report documents a young female patient whose disease course culminated in phthisis bulbi. Through critical analysis of the diagnostic oversights and therapeutic delays encountered in this case, we seek to extract essential clinical lessons that may enhance provider vigilance and inform evidence‐based management strategies for this debilitating disease.

## 2 Case Presentation

This case report was prepared in accordance with the CARE (Case Report) guidelines.

The patient, a 17‐year‐old female of Yi ethnicity from a high‐tuberculosis‐burden area (Liangshan Yi Autonomous Prefecture, Sichuan Province) with a family history of tuberculosis (her cousin), presented on January 6, 2025. She reported with a one‐month history of progressive vision loss, ocular redness, and pain in her right eye. Symptoms had commenced on December 8, 2024, while she was employed in Guangdong Province. She was initially misdiagnosed with “conjunctivitis” at a local clinic and treated with levofloxacin eye drops without improvement. Her condition subsequently deteriorated, prompting referral to Sichuan Eye Hospital with suspected endophthalmitis of unknown origin. She denied any pertinent prior medical or ocular history.

**FIGURE 1:**
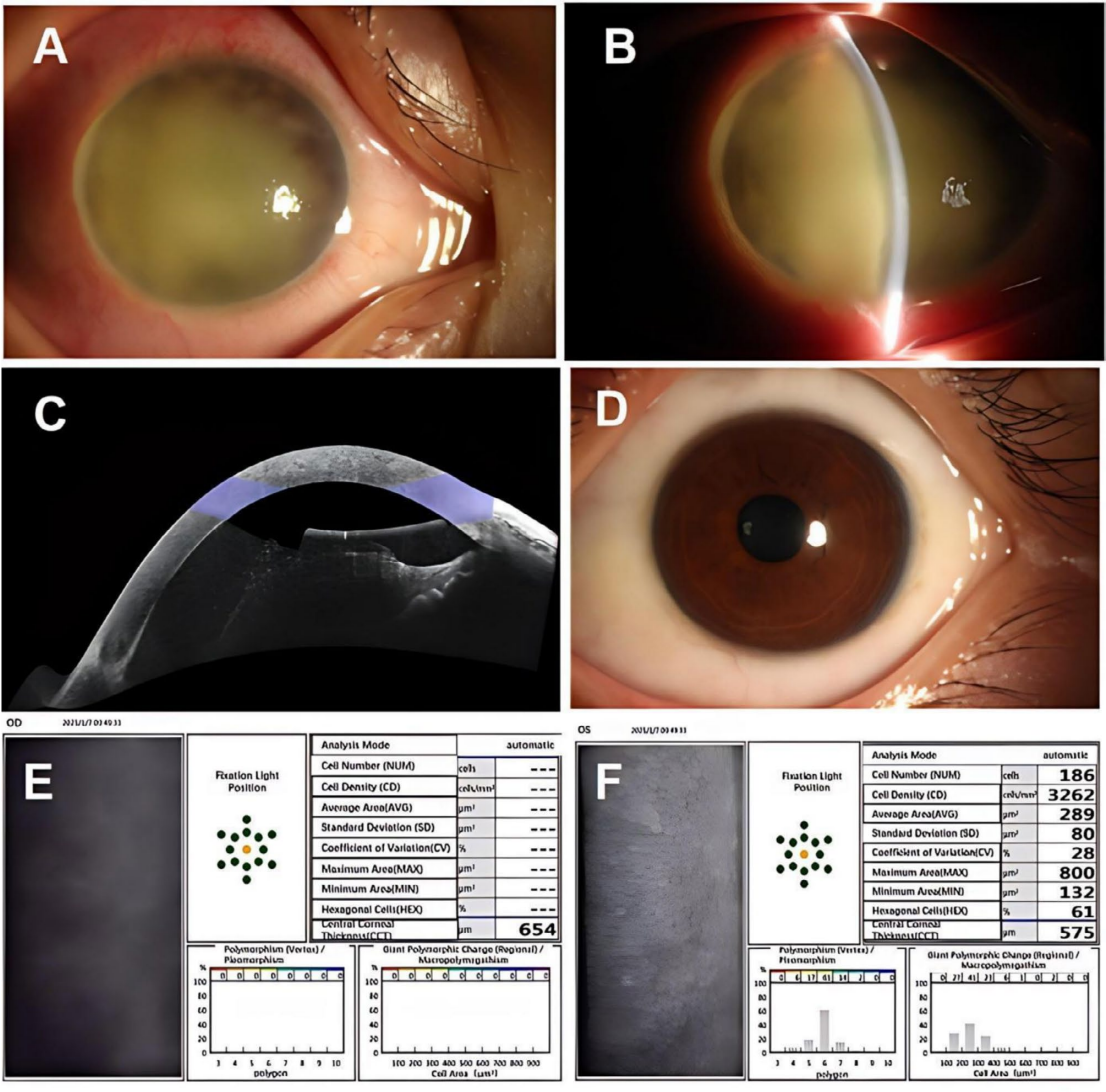
Anterior segment findings. (A) The right eye showed mild mixed conjunctival hyperemia and edema. (B) Slit‐lamp view of the right eye revealed massive white exudates in the anterior chamber. (C) Anterior segment OCT confirmed mild corneal stromal edema with dense exudates on the anterior lens surface. (D) The anterior segment of the left eye was unremarkable. Specular microscopy findings. (E) The right eye showed a central corneal thickness of 645 μm with unrecognizable endothelial cells. (F) The left eye had an endothelial cell density of 3242 cells/mm^2^ and a central corneal thickness of 575 μm.

**FIGURE 2:**
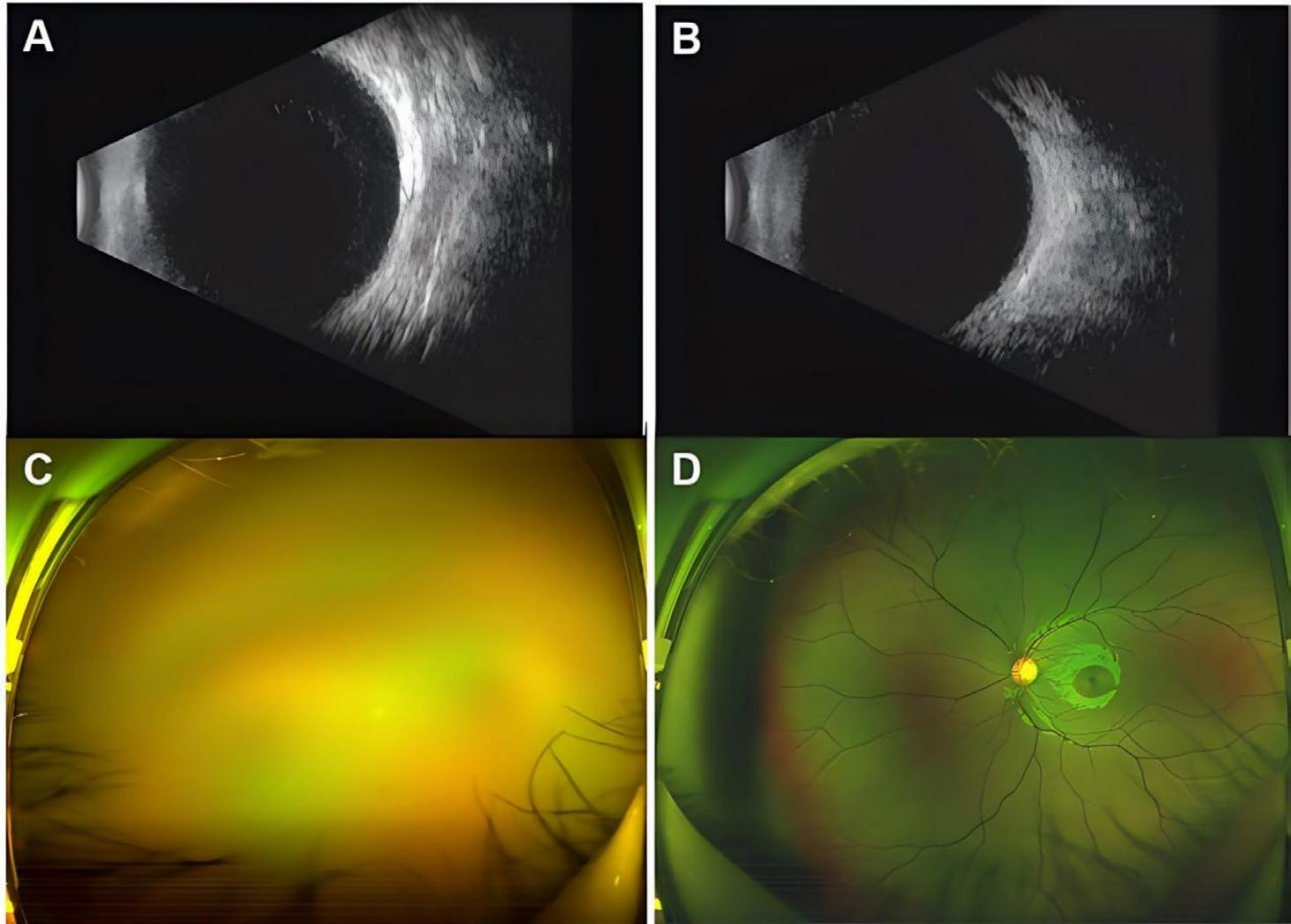
B‐scan ultrasonography and fundus photography findings. (A, B) B‐scan of the right eye revealed moderate vitreous opacity and the left eye showed mild vitreous opacity. (C) Fundus photography of the right eye was obscured. (D) Fundus photography of the left eye showed no obvious abnormalities.

On admission, her vital signs were stable. Ocular examination revealed light perception from the temporal side in the right eye and visual acuity of 1.0 in the left eye. Intraocular pressure (IOP) was 39 mmHg in the right eye and 17 mmHg in the left eye. The right eye showed mild eyelid swelling, mild mixed conjunctival congestion (++), conjunctival edema (+), diffuse punctate corneal epithelial defects with positive fluorescein staining, mild stromal edema, and a large amount of white exudates in the anterior chamber (Figure 1A,B). The posterior segment was not visible. The left anterior segment showed no significant abnormalities (Figure 1D). Anterior segment OCT of the right eye confirmed mild corneal stromal edema and dense, hyperreflective exudates on the anterior lens surface (Figure 1C). Specular microscopy of the right eye revealed central corneal thickness of 645um with indistinct endothelial cells morphology, while the left eye had an endothelial cell density of 3242 cells/mm^2^and central corneal thickness of 575 um (Figure 1E,F). B‐scan ultrasonography demonstrated moderate vitreous opacities in the right eye consistent with severe intraocular inflammation, and mild opacities in the left eye (Figure 2A,B). Fundus photography of the right eye was obscured, while the left was normal (Figure 2C,D). Systemic laboratory investigations on admission revealed a white blood cell count of 5.07 × 10^9^/L, with an eosinophil percentage of 11% (normal < 5%), a lymphocyte count of 1.23 × 10^9^/L (normal 1.1–3.2), and a lymphocyte percentage of 24.3%. Liver and kidney function tests showed elevated globulin at 40.2 g/L (normal 20–35) and a decreased albumin/globulin ratio of 1.08 (normal 1.2–2.4), with a direct bilirubin of 5.0 μmol/L (normal 0–3.4). Screening for HIV, hepatitis B, hepatitis C, and syphilis were all negative.

**FIGURE 3:**
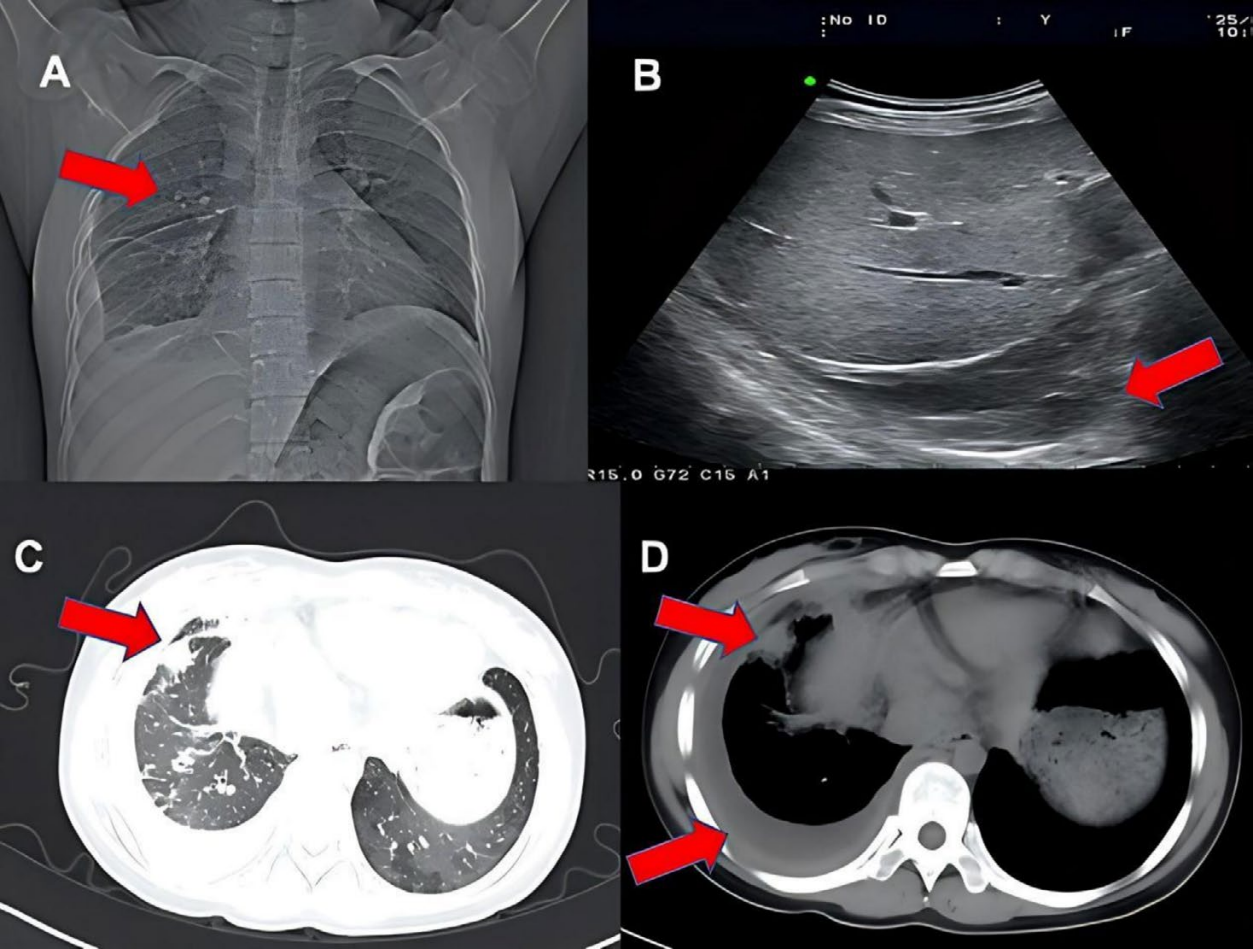
Systemic imaging findings. (A) Chest radiograph showed a band‐like dense opacity in the lateral chest wall and right pleural thickening with a small effusion. (B) Abdominal ultrasound revealed a free anechoic area in the right pleural cavity. (C, D) Chest CT demonstrated diffusely distributed multiple small nodules in both lungs with scattered patchy infiltrates and enlarged mediastinal and right hilar lymph nodes, consistent with miliary pulmonary tuberculosis.

Systemic imaging included a cranial CT scan that excluded intraocular foreign bodies or tumors. A chest X‐ray showed a right‐sided pleural thickening and small effusion (Figure 3A). Abdominal ultrasound detected a 22 mm pleural fluid area in the right hemithorax (Figure 3B). Chest CT scan was definitive, revealing diffuse miliary nodules, patchy infiltrates, and mediastinal/hilar lymphadenopathy consistent with miliary tuberculosis (Figure 3C,D).

An anterior chamber paracentesis was performed. Due to laboratory limitations at our specialized eye hospital, PCR testing required sending the sample to an external institution, with a turnaround time of over 1 week and significant cost, which the patient and her family declined. Consequently, only smear and culture were performed. The aqueous humor sample was negative for acid‐fast bacilli, fungi, and routine bacteria cultures. Based on these strongly suggestive radiography findings, a multidisciplinary team (MDT) discussion considered a diagnosis of miliary pulmonary tuberculosis. Given the high infectivity and life‐threatening nature of the systemic disease, and concerns from our infection control department about nosocomial transmission, the patient was transferred to a specialized infectious disease hospital 2 days later (January 8, 2025) for systemic management. Further tests confirmed the diagnosis: a positive PPD test (++), positive T‐SPOT.TB test, and most importantly, a positive Xpert MTB/RIF assay on bronchoalveolar lavage fluid, which also showed sensitivity to rifampicin. A diagnosis of pulmonary tuberculosis was established, and a standard four‐drug anti‐tuberculosis therapy (2HREZ/4HR regimen) was initiated on 5 days later (January 11, 2025).

### 2.1 Differential Diagnosis

**FIGURE 4:**
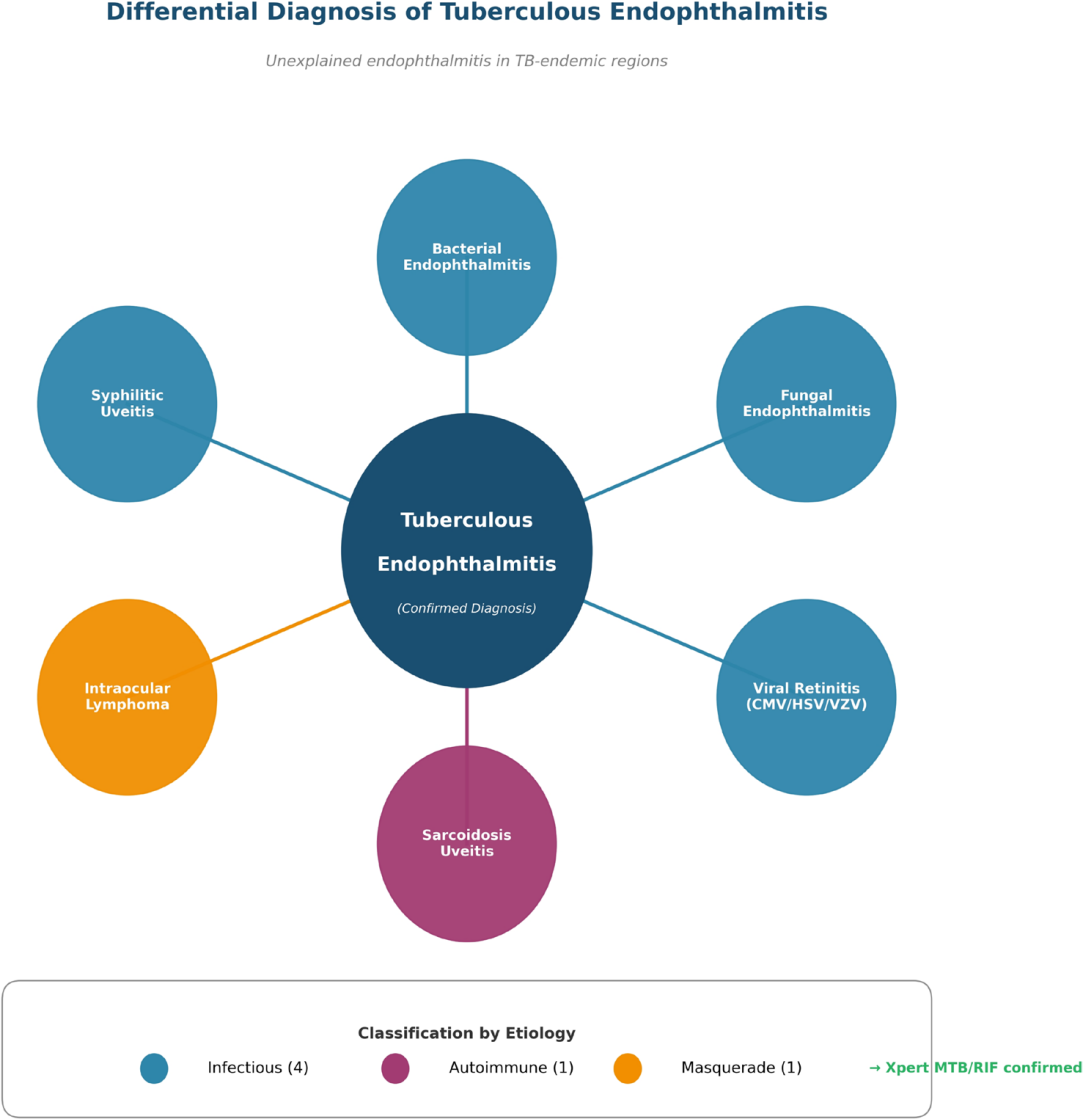
Differential diagnosis of tuberculous endophthalmitis. The diagram illustrates six major diagnostic considerations categorized by etiology: infectious (blue), autoimmune (purple), and masquerade (orange). The final diagnosis of tuberculous endophthalmitis was confirmed by positive Xpert MTB/RIF assay from bronchoalveolar lavage fluid.

The differential diagnosis of unexplained endophthalmitis in tuberculosis‐endemic regions requires systematic evaluation across infectious, autoimmune, and masquerade etiologies (Figure 4).

The diagnostic reasoning proceeded through hierarchical exclusion:

### 2.2 Infectious Etiologies

*Bacterial endophthalmitis* typically presents with acute onset, severe pain, and hypopyon formation. However, this patient's subacute one‐month course and granulomatous inflammatory features were atypical for bacterial infection.

*Fungal endophthalmitis* was considered given the white exudates observed in the anterior chamber. Nevertheless, the absence of systemic risk factors (immunocompromise, intravenous drug use) and negative fungal cultures argued against this diagnosis.

*Viral retinitis* (CMV, HSV, VZV) generally occurs in immunocompromised hosts and demonstrates characteristic necrotizing retinitis patterns. Our patient's HIV‐negative status and absence of retinal necrosis on imaging made this unlikely.

*Syphilitic uveitis* can present with granulomatous inflammation and placoid chorioretinitis. However, negative syphilis serology effectively excluded this diagnosis.

### 2.3 Autoimmune Etiology

*Sarcoidosis‐associated panuveitis* shares granulomatous features with tuberculous disease. The absence of hilar lymphadenopathy on chest CT and lack of elevated serum ACE levels, combined with the definitive evidence of miliary tuberculosis, made sarcoidosis improbable.

### 2.4 Masquerade Syndrome

*Intraocular lymphoma* typically presents in older patients with subacute vitreous haze and subretinal infiltrates. The patient's young age (17 years), subacute inflammatory presentation, and systemic findings of miliary tuberculosis rendered lymphoma clinically unlikely.

### 2.5 Diagnostic Confirmation

The final diagnosis of tuberculous endophthalmitis secondary to miliary pulmonary tuberculosis was established by positive Xpert MTB/RIF assay from bronchoalveolar lavage fluid, which demonstrated rifampicin‐sensitive *Mycobacterium tuberculosis* complex. This molecular confirmation, combined with the characteristic systemic radiographic findings (diffuse miliary nodules, patchy infiltrates, mediastinal lymphadenopathy) and epidemiological context (residence in high‐burden region, family contact history), provided definitive diagnostic clarity.

## 3 Conclusion and Results (Outcome and Follow‐Up)

**FIGURE 5:**
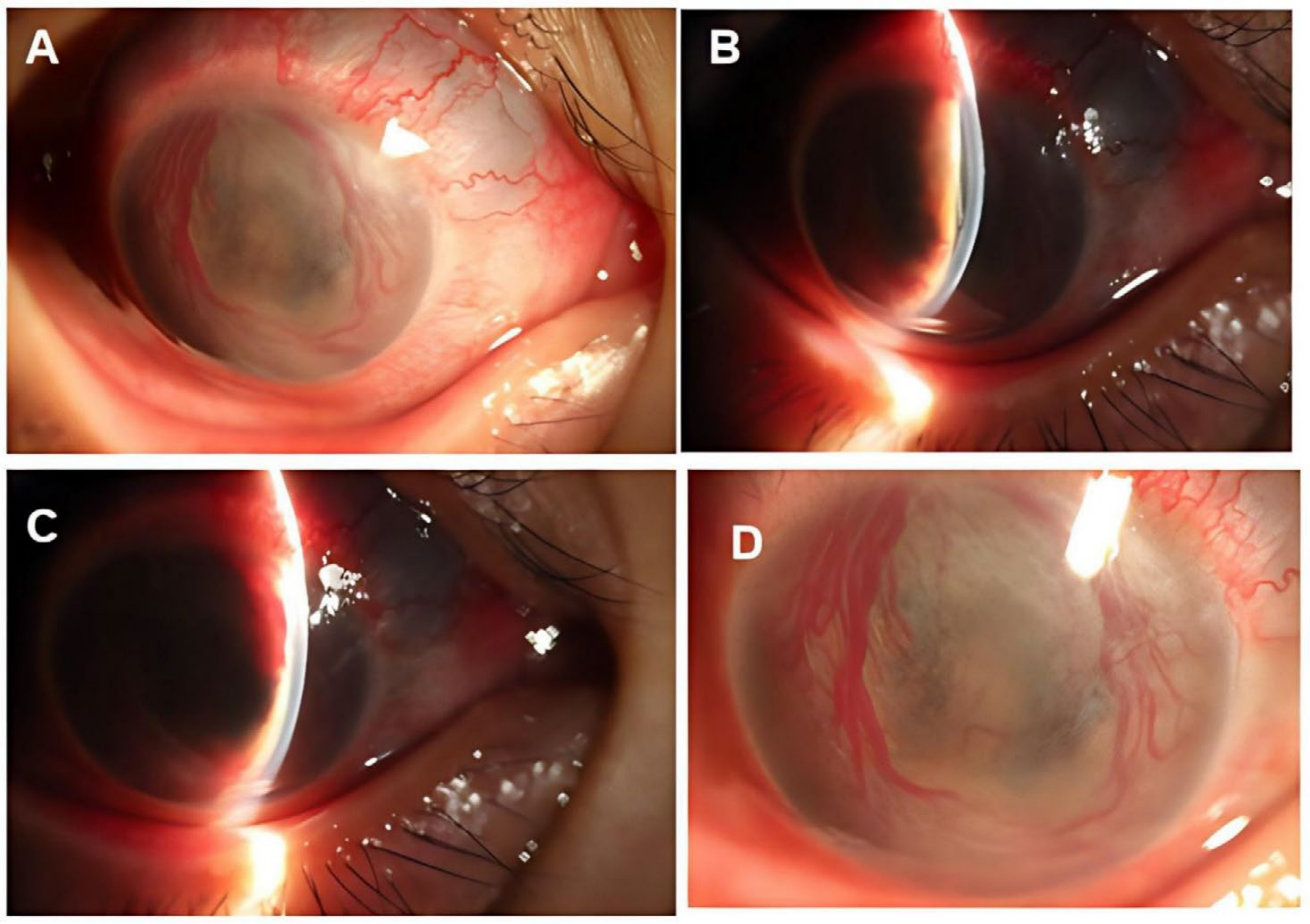
Anterior segment photography at one‐month follow‐up. (A, B) The right eye showed mixed conjunctival hyperemia and peripheral corneal neovascularization, the anterior chamber was shallow with extensive iris‐cornea apposition. (C, D) Coarse, tortuous iris neovascularization with superior hemorrhage was visible.

One month after starting anti‐tuberculosis therapy, the right eye IOP was 18 mmHg. The cornea was thickened with peripheral neovascularization, extensive peripheral anterior synechiae, and iris neovascularization (rubeosis iridis) with concomitant hemorrhage (Figure 5A–D).

**FIGURE 6:**
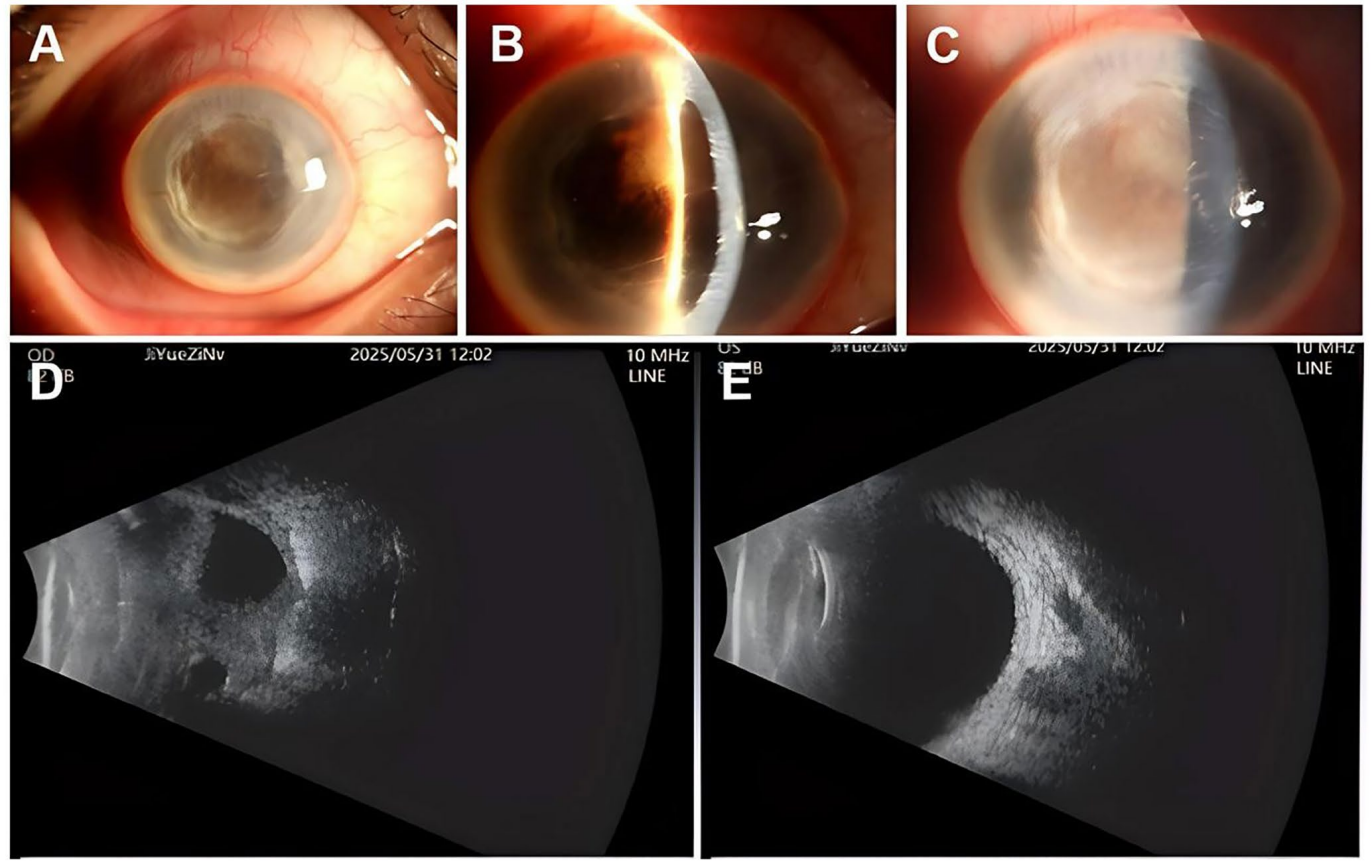
Anterior segment photography and B‐scan ultrasonography at 6‐month follow‐up. (A) The right eye showed conjunctival hyperemia. (B) The cornea was thickened with a central anterior chamber depth of 2 CT. (C) Extensive peripheral anterior synechiae and iris neovascularization (rubeosis iridis) with concomitant hemorrhage. (D) The right eye showed disorganized intraocular structures and a shortened axial length, consistent with phthisis bulbi. (E) The left eye showed persistent mild vitreous opacity.

At the six‐month follow‐up, the right eye was quiet with an IOP of 11 mmHg, but vision remained light perception from the temporal side. The pupil was secluded, and peripheral anterior synechiae persisted (Figure 6A–C). B‐scan ultrasonography confirmed phthisis bulbi, with a shortened axial length (22.47 mm) and disorganized intraocular structures (Figure 6D,E). The left eye remained unaffected throughout the treatment course.

## 4 Discussion and Conclusion

This case of tuberculous endophthalmitis in a young woman, culminating in phthisis bulbi, serves as a powerful cautionary tale. Its value lies not in a successful outcome, but in the critical lessons gleaned from its diagnostic and therapeutic trajectory.

First, insufficient disease awareness and clinical vigilance. The patient originated from a high tuberculosis‐burden region (Liangshan, Sichuan) and had a direct family contact history, both critical clues that were not adequately weighted in the initial diagnostic phase [5, 6]. Her presentation with subacute, granulomatous panuveitis accompanied by severe vitreous opacification and secondary ocular hypertension—features consistent with tuberculous panuveitis [7, 8]. However, during the initial diagnostic phase, clinical reasoning remained narrowly focused on ocular findings, failing to promptly correlate the ocular manifestations with epidemiological history and potential systemic disease, resulting in a misdirected diagnostic approach.

Second, delayed and inadequate diagnostic and therapeutic strategies highlight systemic and therapeutic missteps. This case underscores two pivotal errors:

- Neglect of systemic evaluation and failure of timely referral: Ocular involvement frequently constitutes the inaugural manifestation of systemic tuberculosis [9]. In this instance, chest CT proved the decisive step in unveiling the underlying etiology. For any case of severe endophthalmitis of unknown origin, particularly within tuberculosis‐endemic regions, chest imaging should constitute an urgent routine screening measure rather than an exclusive last‐resort investigation. More critically, after diagnosing miliary tuberculosis, the decision was made to transfer the patient to an infectious disease hospital for systemic control, prioritizing the life‐threatening systemic condition and infection control within our specialized eye hospital. While understandable, this led to a failure to concurrently arrange referral to a tertiary center with both infectious disease and vitreoretinal surgical capabilities. This fragmentation of care, driven by institutional limitations, directly resulted in the missed opportunity for a diagnostic and therapeutic pars plana vitrectomy (PPV), which could have secured a definitive ocular diagnosis and reduced the inflammatory and infectious load [10]. It should be noted that while PPV may increase diagnostic yield and reduce infectious load in selected cases, the available evidence supporting its routine use in tuberculous endophthalmitis remains limited [10].
- Suboptimal therapeutic regimen: The management of TE requires a dual approach. While systemic anti‐tuberculosis therapy (ATT) is the cornerstone [11, 12], the management at the infectious disease hospital was overly conservative, withholding adjunctive corticosteroids. This decision was likely driven by concerns of exacerbating a severe, disseminated infection.
- However, current evidence and guidelines suggest that systemic corticosteroids, initiated 1–2 weeks after effective ATT, may help suppress immune‐mediated inflammation in ocular TB [12, 13]. However, the role of corticosteroids in severe tuberculous endophthalmitis specifically is not well established, and their use must be balanced against the risk of exacerbating disseminated infection. The absence of timely steroid administration in this patient may have contributed to the rapid progression to phthisis bulbi, though the aggressive natural course of severe endogenous tuberculous endophthalmitis must also be acknowledged as a significant factor [3, 4].

Finally, the catastrophic outcome resulted from the synergistic effect of a one‐month delay in patient presentation and subsequent healthcare process delays. It profoundly cautions that when confronting severe or enigmatic endophthalmitis, the most responsible approach is prompt referral to a tertiary center with comprehensive expertise.

Pathophysiological Considerations Intraocular inflammation associated with tuberculosis may reflect direct infection by *Mycobacterium tuberculosis* , immune‐mediated mechanisms, or both [1, 2]. In the present case, the exact mechanism remains inferential in the absence of microbiologic confirmation from ocular samples. The severe destructive course observed is consistent with the known aggressive nature of endogenous tuberculous endophthalmitis, which often leads to poor visual outcomes despite appropriate therapy [3, 4].

In summary, tuberculosis‐endemic regions, tuberculous endophthalmitis should be prioritized as a principal differential diagnosis for unexplained endophthalmitis. Immediate systemic evaluation and timely intervention are crucial for preserving vision and preventing globe atrophy. Ophthalmologists must adopt an integrated ocular‐systemic approach, with chest imaging and comprehensive systemic evaluation forming the cornerstone of early diagnostic workup. For highly suspicious cases, prompt diagnostic pars plana vitrectomy may be considered, though evidence for its efficacy remains limited. Adjunctive corticosteroids should be initiated cautiously, with careful consideration of risks and benefits. In resource‐limited settings, establishing efficient referral pathways to appropriate tertiary care centers is critical to prevent permanent vision loss and globe atrophy.

## Author Contributions

**Yanchen Chen:** conceptualization, writing – original draft, writing – review and editing. **Shengnan Li:** visualization. **Xin Chen:** validation. **Ningpu Liu:** supervision. **Dongjing Liu:** conceptualization, funding acquisition, resources.

## Funding

This work was supported by the Chengdu Municipal Health Commission (2022500).

## Ethics Statement

Ethics approval for this case report was obtained from the Institutional Review Board of Sichuan Eye Hospital, AIER Eye Hospital Group. The study adhered to the tenets of the Declaration of Helsinki.

## Consent

Written informed consent for the publication of this case report and any accompanying images was obtained from the patient's legal guardian, as the patient was a minor at the time of initial treatment.

## Conflicts of Interest

The authors declare no conflicts of interest.

## Data Availability

The data that support the findings of this study are available on request from the corresponding author. The data are not publicly available due to privacy or ethical restrictions.
